# Stevens Johnson Syndrome in a Patient with Giant Cell Arteritis During Short Term Tocilizumab Therapy

**DOI:** 10.7759/cureus.7662

**Published:** 2020-04-13

**Authors:** Niranjani Venkateswaran, Reena Khianey, August Generoso

**Affiliations:** 1 Internal Medicine, Penn State Health Milton S. Hershey Medical Center, Hershey, USA; 2 Allergy and Immunology, Rutgers New Jersey Medical School, Newark, USA

**Keywords:** giant cell arteritis, human leukocyte antigen, stevens johnson syndrome, tocilizumab, natural killer, t-helper, tumor growth factor

## Abstract

This case report represents a rare life-threatening hypersensitivity reaction of tocilizumab drug when it is used to treat giant cell arteritis. An elderly female with history of bilateral giant cell arteritis with anterior ischemic optic neuropathy of the right eye was started on tocilizumab after developing glucocorticoid-related complications. She received one month of the tocilizumab therapy along with the prednisone taper. The patient initially developed sinus and mucosal edema, presented as drooling with mild tongue and lip swelling. It eventually progressed into development of new onset of erythematous macules and flaccid bullae which was biopsy-confirmed Stevens Johnson syndrome. Tocilizumab drug was immediately discontinued and she was treated with supportive care. The goal of this report is to present the first detailed case of presumed tocilizumab-induced Steven Johnson syndrome which emphasizes the importance of post-marketing surveillance and collection of data on adverse events of this drug.

## Introduction

Tocilizumab (TCZ) is a monoclonal antibody against interleukin 6 (IL-6) receptor. Inhibition of IL-6 signaling by TCZ has been found to be effective in the treatment of rheumatoid arthritis (RA) and systemic juvenile idiopathic arthritis (sJIA). More recently, a large study confirmed the efficacy of TCZ as a glucocorticoid sparing option in giant cell arteritis (GCA). The findings showed that TCZ combined with a prednisone taper was found to be superior in causing GCA remission compared to prednisone alone [1]. Adverse reactions of TCZ include infections, hepatotoxicity, neutropenia, thrombocytopenia, hyperlipidemia, and hypersensitivity reactions [2]. We report the first detailed case of Stevens Johnson syndrome (SJS) presumably due to TCZ in a patient with underlying autoimmune disease.

## Case presentation

**Figure 1:**
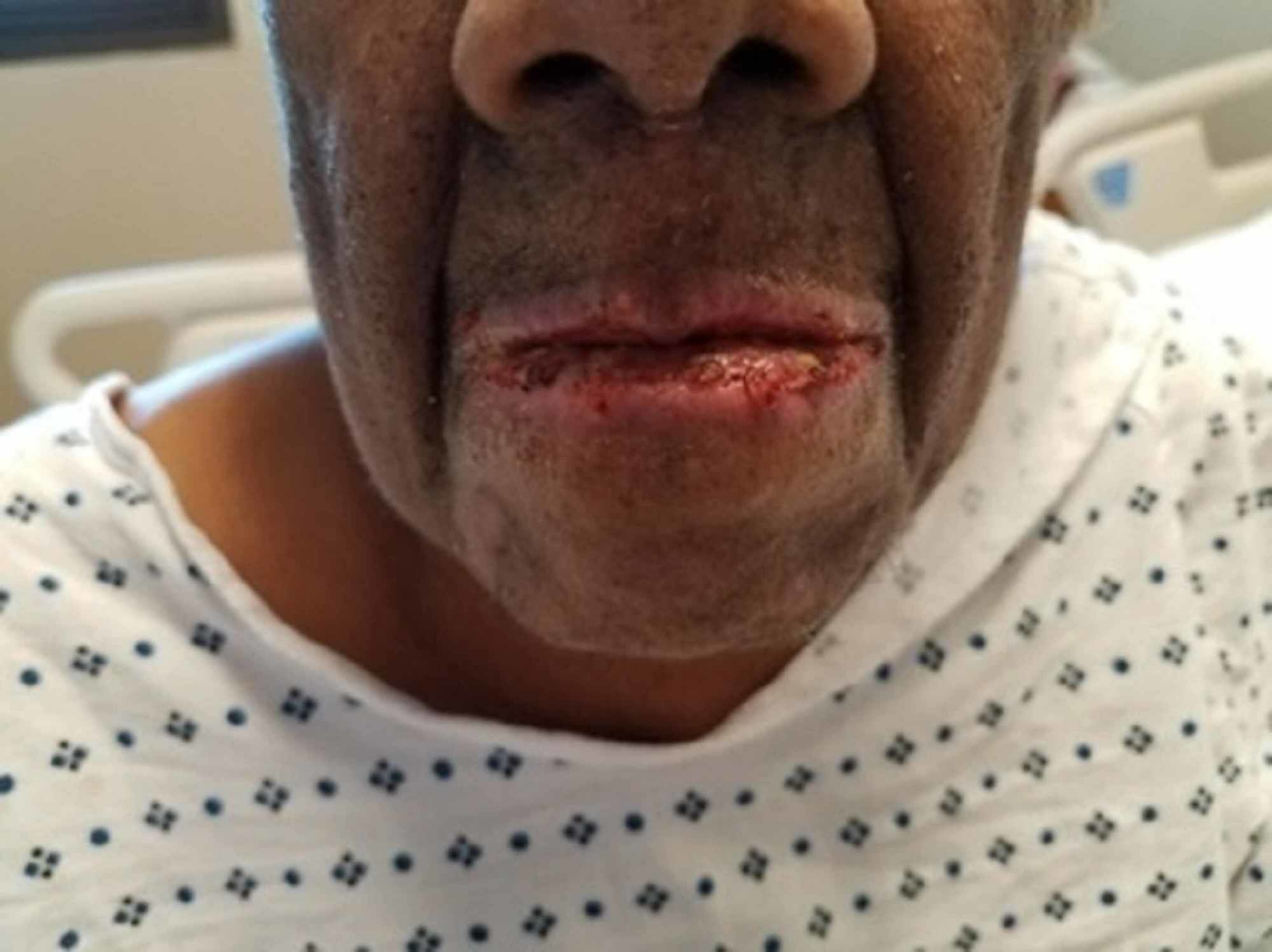
Development of mucosal lesions in Steven Johnson Syndrome

**Figure 2:**
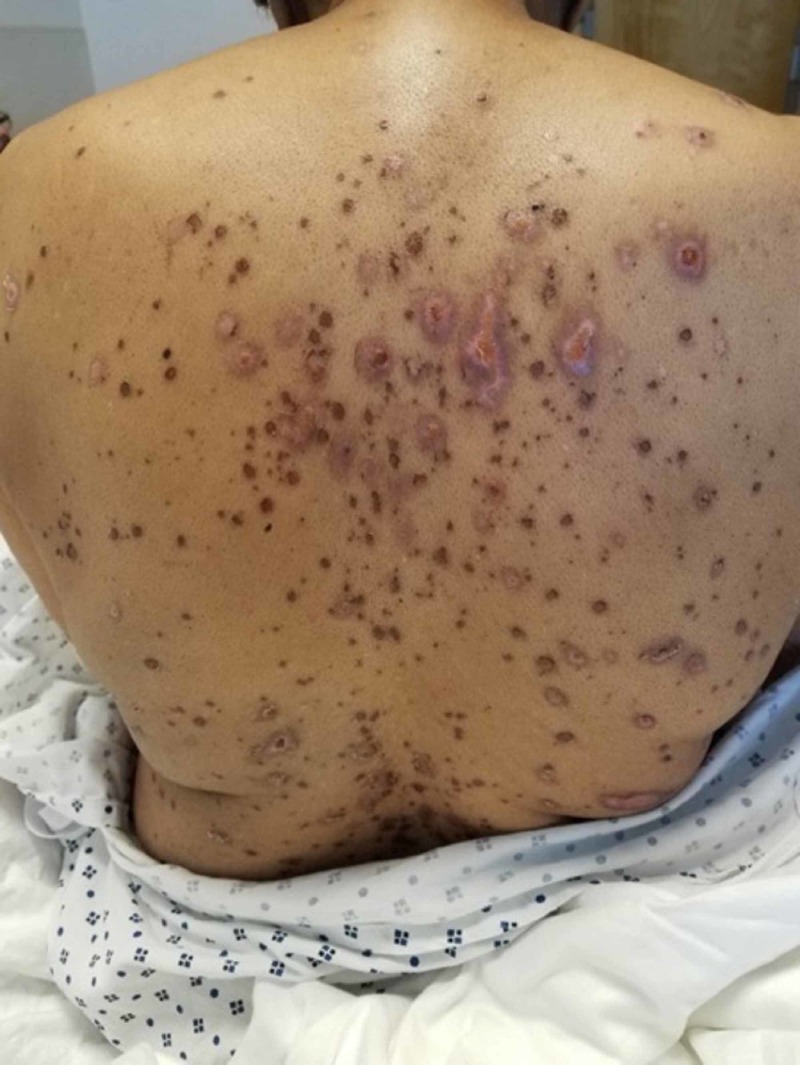
Erythematous flaccid/bullous lesions in Steven Johnson Syndrome

An 82-year-old African-American woman with a past medical history of diabetes mellitus type II, hypertension, hypothyroidism, and biopsy-confirmed bilateral GCA with anterior ischemic optic neuropathy of the right eye was started on TCZ after developing glucocorticoid-related complications. She was started on 162 milligrams of TCZ subcutaneously once per week in addition to a prednisone taper. After one month of therapy, the patient presented to our emergency department (ED) with complaints of pharyngitis and odynophagia. One day prior to onset of symptoms, she received topical proparacaine eye drops during a routine ophthalmologic visit. She denied any new medications or exposures to any new products. Home medications included alendronate, aspirin, atovaquone, calcium-carbonate-vitamin D, glipizide, metformin, levothyroxine, and olmesartan-amlodipine-hydrochlorothiazide. In the ED, physical examination was significant for drooling and mild tongue and lip swelling, with open sores on oral commissures bilaterally. Inflammatory markers (erythrocyte sedimentation rate and C-reactive protein) were within normal limits. A CT scan of the sinuses displayed edema and mucosal thickening of the wall of nasopharynx and oropharynx. Due to concern for an allergic reaction with possible airway compromise, she was treated with IV steroids and admitted for further observation. Hospital course was complicated by progressive dysphagia, conjunctivitis and mucosal ulcers (Figure 1). She also developed new onset erythematous macules on her back and flaccid bullae on her palms, back, and extremities, involving 8% of her skin (Figure 2). She was diagnosed with Stevens Johnson syndrome (SJS), which was further supported by skin biopsy demonstrating epidermal necrosis. Her SJS was presumptively due to TCZ, which was thus discontinued. She was transferred to the ICU and managed with IV fluids, steroids, and intravenous gamma globulin (IVIG). She responded well to treatment with no new lesions and resolution of existing lesions after two weeks of supportive care.

## Discussion

SJS is an acute life-threatening hypersensitivity reaction involving the skin and mucous membranes. The disease is characterized by extensive necrosis and detachment of the epidermis, leading to blisters and mucosal membrane erosions. The most common causes of SJS are medications followed by infections. SJS usually develops within four weeks of starting a new medication which includes anticonvulsants, antibiotics, analgesics, and immunosuppressants. The underlying pathogenesis is incompletely understood but involves interaction with human leukocyte antigen (HLA) leading to T cell- and natural killer (NK) cell-mediated apoptosis of keratinocytes. The disease usually begins with a prodrome of fever and flu-like symptoms followed by skin lesions within three days. The lesions appear as erythematous macules, which eventually progress into vesicles/bullae and sloughs off within days. Mucosal involvement occurs in 90% of cases and can be oropharyngeal, ocular, or urogenital. Management involves immediate discontinuation of the suspected drug and supportive care. Adjunctive therapies include steroids and/or IVIG, though data regarding their effects on SJS are conflicting [3,4]. Based on our literature search, there is only one unconfirmed case of SJS due to TCZ; this was mentioned in the trial but the details were not described [5]. We present the first detailed case of presumed TCZ-induced SJS, highlighting the need for post-marketing surveillance and collection of data on adverse events of this drug.

Unique to our patient is that she had a relatively mild course of the disease. She started developing the symptoms after three doses of tocilizumab. She did not have a prodromal period nor did she experience any severe constitutional symptoms. We postulate that her concurrent systemic steroid treatment for her GCA played a protective role in her presentation. We also question whether our patient was actually more susceptible to developing SJS because of her underlying GCA or anti-IL-6 therapy. A number of factors are upregulated in SJS patients, including IL-15, IL-8, TNF alpha, and IL-6 [6]. IL-6 is a cytokine that plays a critical role in the balance between pro-inflammatory Th17 cells and suppressive T regulatory cells [7]. Th17 cells are responsible for inducing a number of pro-inflammatory cytokines like IL-21, which induces production of granulysin, a cytotoxic protein implicated in the keratinocyte apoptosis that occurs in SJS. Of note, GCA itself is known to have Th17 upregulation as well [8-10]. Along with TGF-beta, IL-6 can restrain this inflammatory response of activated Th17 cells by inducing IL-10 production. With this in mind, we propose that our patient may potentially have been more susceptible to developing SJS, either due to her underlying GCA which can increase IL17/21 signaling and/or due to TCZ’s reduction of the anti-inflammatory effects of IL-6.

## Conclusions

Based on our case presentation, TCZ can increase the risk of developing SJS, especially in patients with underlying GCA due to the upregulation of pro-inflammatory cytokine Th17 in the setting of IL-6 suppression. This report emphasizes the importance of post-marketing surveillance and other adverse effects of tocilizumab, specifically in patients with an underlying autoimmune disorder.
